# Study on the Relationship between the miRNA-centered ceRNA Regulatory Network and Fatigue

**DOI:** 10.1007/s12031-021-01845-3

**Published:** 2021-05-16

**Authors:** Xingzhe Yang, Feng Li, Jie Ma, Yan Liu, Xuejiao Wang, Ruochong Wang, Yifei Zhang, Wei Zhang, Qingyun He, Dandan Song, Jiaojiao Yu

**Affiliations:** College of Traditional Chinese Medicine, Beijing University of Chinese Medicine, Beijing, China

**Keywords:** MiRNA, ceRNA, NF-κB pathway, Fatigue, lncRNA, circRNA

## Abstract

In recent years, the incidence of fatigue has been increasing, and the effective prevention and treatment of fatigue has become an urgent problem. As a result, the genetic research of fatigue has become a hot spot. Transcriptome-level regulation is the key link in the gene regulatory network. The transcriptome includes messenger RNAs (mRNAs) and noncoding RNAs (ncRNAs). MRNAs are common research targets in gene expression profiling. Noncoding RNAs, including miRNAs, lncRNAs, circRNAs and so on, have been developed rapidly. Studies have shown that miRNAs are closely related to the occurrence and development of fatigue. MiRNAs can regulate the immune inflammatory reaction in the central nervous system (CNS), regulate the transmission of nerve impulses and gene expression, regulate brain development and brain function, and participate in the occurrence and development of fatigue by regulating mitochondrial function and energy metabolism. LncRNAs can regulate dopaminergic neurons to participate in the occurrence and development of fatigue. This has certain value in the diagnosis of chronic fatigue syndrome (CFS). CircRNAs can participate in the occurrence and development of fatigue by regulating the NF-κB pathway, TNF-α and IL-1β. The ceRNA hypothesis posits that in addition to the function of miRNAs in unidirectional regulation, mRNAs, lncRNAs and circRNAs can regulate gene expression by competitive binding with miRNAs, forming a ceRNA regulatory network with miRNAs. Therefore, we suggest that the miRNA-centered ceRNA regulatory network is closely related to fatigue. At present, there are few studies on fatigue-related ncRNA genes, and most of these limited studies are on miRNAs in ncRNAs. However, there are a few studies on the relationship between lncRNAs, cirRNAs and fatigue. Less research is available on the pathogenesis of fatigue based on the ceRNA regulatory network. Therefore, exploring the complex mechanism of fatigue based on the ceRNA regulatory network is of great significance. In this review, we summarize the relationship between miRNAs, lncRNAs and circRNAs in ncRNAs and fatigue, and focus on exploring the regulatory role of the miRNA-centered ceRNA regulatory network in the occurrence and development of fatigue, in order to gain a comprehensive, in-depth and new understanding of the essence of the fatigue gene regulatory network.

## Background

With the accelerated pace of life, fatigue has become an important factor affecting people's quality of life. One survey report found that more than half of the population feels tired, and more than a third clearly stated that fatigue had greatly reduced the quality of life and work efficiency (Watanabe 2008). The causes of fatigue are complex and can have serious consequences. Fatigue can be a symptom that accompanies other diseases, such as malignant tumors, multiple sclerosis, iron deficiency anemia, stroke or Parkinson's disease. It can be an independent disease. It is also a common side effect of anticonvulsants, analgesics, antidepressants, etc. (Li et al. 2016) The World Health Organization has listed fatigue as one of the main factors endangering human health in the twenty-first century. The effective prevention and treatment of fatigue has become an urgent problem.

### mRNA is a Common Research Object of the Fatigue Gene Expression Profile

Since the beginning of the twenty-first century, various scholars have proposed that fatigue has genetic susceptibility. Since then, increasing evidence shows that fatigue is closely related to gene changes (Helliwell et al. 2020; Lievesley et al. 2014), and the gene set of upregulated and downregulated genes in chronic fatigue has been revealed (Norheim et al. 2011; Frampton et al. 2011; Nguyen et al. 2017; Rajeevan et al. 2015). Gene chip research of the fatigue gene expression profile has been carried out.

Ribonucleic acids (RNAs) are essential polymeric molecules in many biological processes, such as encoding, decoding, regulating and expressing genetic information. It is one of the four major macromolecules that constitute all known life forms. Messenger RNAs (mRNAs) transfer genetic information from deoxyribonucleic acid (DNA) to ribosomes. The coding sequence of mRNA determines the type and order of amino acids in protein, which is the "bridge" of biological information transmission between DNA and proteins (Costa et al. 2010). Therefore, mRNAs are common genetic research objects.

### MiRNAs are Closely Related to the Occurrence and Development of Fatigue

MiRNAs are 17–25 nucleotides long and control gene expression at the post-transcriptional level (Matsuyama and Suzuki 2019; Ha et al. 2014; Pu et al. 2019). Under certain conditions, miRNAs can also activate targeted mRNAs (Correia et al. 2019; Stavast et al. 2019). MiRNA can regulate the expression of mitochondrial-related genes and affect the morphology and function of mitochondria which play an important role in the process of energy metabolism (Konovalova et al. 2019; He et al. 2014). Studies have shown that damage to mitochondrial DNA (mtDNA) leads to mitochondrial dysfunction and energy metabolism disorders, which is the underlying pathophysiological mechanism of chronic fatigue syndrome (Yamano et al. 2016; El-Hattab et al. 2017). Some other scholars have confirmed through experiments that the decrease of oxygen metabolism in the central nervous system can lead to the limitation of muscle activity and fatigue (Halley et al. 2019; Rupp et al. 2015). Thus miRNAs are involved in the regulation of mitochondrial function and energy metabolism and affect the fatigue of the body. Therefore, mRNAs are commonly targeted for genetic research.

In recent years, a large number of studies in China and elsewhere have shown that immune inflammation is an important mechanism of fatigue (Capuron and Miller 2011; Haroon et al. 2012). MiRNAs can be used as key regulators of inflammatory response in the central nervous system (CNS) (Slota and Booth 2019). MiRNAs participate in immune response, regulate cytokines and participate in the development and differentiation of lymphocytes (Soltanzadeh et al. 2018; Leung et al. 2018). MiRNAs are involved in the development of chronic fatigue syndrome (CFS) by regulating immune inflammatory response (Brenu et al. 2014). Some studies have shown that the differential expression of miRNAs in peripheral blood mononuclear cells (PBMC) of CFS patients can regulate the function of immune cells, such as T cells, B cells, NK cells and macrophages, and participate in immune and inflammatory reactions, thus affecting the occurrence and development of fatigue (Petty et al. 2016; Brenu et al. 2012).

A study using gene chip technology found that 34 kinds of miRNAs were overexpressed in the peripheral blood of patients with chronic fatigue syndrome. Among them, the expression of hsa-mir-99b and hsa-mir-330-3p was upregulated in B cells and NK cells. These two miRNAs can be transfected into NK cells, resulting in gene expression changes, NK cell activation and cytotoxicity reduction, which leads to immune dysfunction (Petty et al. 2016). In another study, researchers measured the miRNAs in NK cells and CD8^+^ T cells of CFS patients, and found that the expression levels of miR-21 in NK cells and CD8^+^ T cells, and miR-17-5p, miR-10a, miR-103, miR-152, miR-146a, miR-106, miR-223 and mi R-191 in NK cells were significantly lower than those in the normal control group. These miRNAs are involved in cell apoptosis, cell cycle and immune response, and thus affect cell function (Brenu et al. 2012). Screening of peripheral blood samples of patients with clinically diagnosed CFS and comparison with age-matched healthy controls revealed that hsa-miR-99b, hsa-miR-330-3p, hsa-miR-30c and hsa-miR-126 in NK cells can become effective biomarkers for CFS.

MiRNAs also play an important role in the regulation of gene expression and participate in the occurrence and development of fatigue. MiRNA expression disorder can cause a variety of human diseases, such as tumors and cardiovascular, metabolic, rheumatic and neurological diseases (Bhayani et al. 2012). Studies of Cui (Cui et al. 2016) have confirmed that miRNAs, such as let-7b-5P, miR-148a-3P, miR-124-3P, miR-107-3P and miR-370-3P, can regulate the transmission of nerve impulses and gene expression, regulate the tolerance of the hypothalamus to acupuncture treatment and affect the fatigue of the body.

**Fig. 1:**
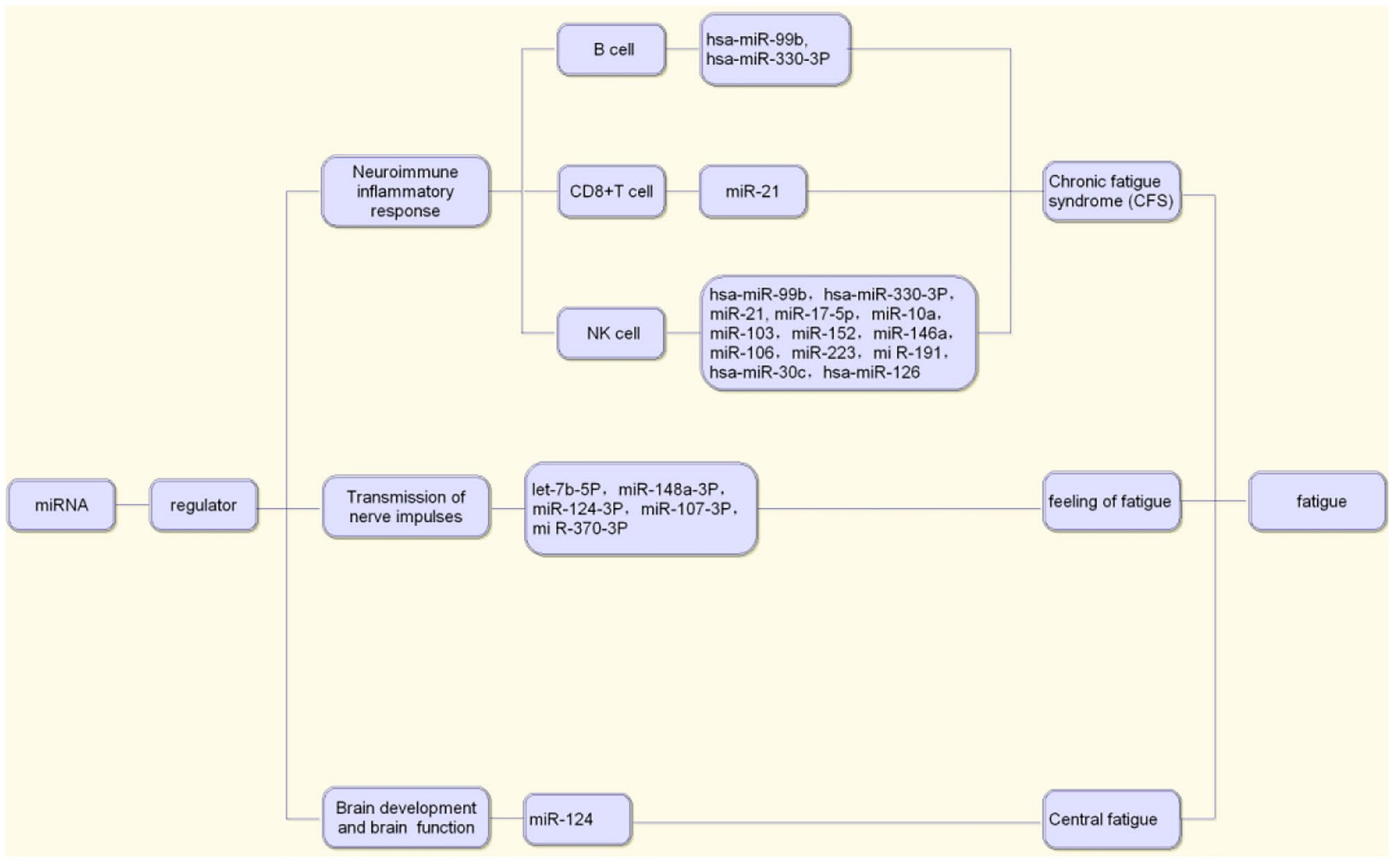
MiRNAs associated with fatigue

Due to the quickening pace of life and the increasing mental stress in modern society, central fatigue has garnered increasing attention. According to Leavitt (Leavitt and DeLuca 2010), the core mechanism of central fatigue is closely related to the impairment of nerve pathway conduction function and abnormal regulation of neurotransmitters. MiRNAs play an important role in brain development and brain function. The loss of the enzyme Dicer in a developing cerebral cortex leads to the decrease of specific miRNAs, apoptosis of new neurons, thinning of the cerebral cortex and decrease of dendritic branches, which indicates that miRNA plays an important role in brain development. For example, miR-124 enriched in the brain begins on day 13 of the embryo (E13) and remains highly expressed throughout adulthood. It can promote the differentiation of neural progenitor cells into neurons and is inhibited by the repressor element-1-silencing transcription (REST). Therefore, the REST-miR-124 axis plays an important role in the control of neuronal phenotype (Follert et al. 2014; Petri et al. 2014). In conclusion, based on the important role of miRNAs in regulating brain development and brain function, we speculate that miRNAs are involved in the occurrence and development of central fatigue (Fig. 1).

**Fig. 2:**
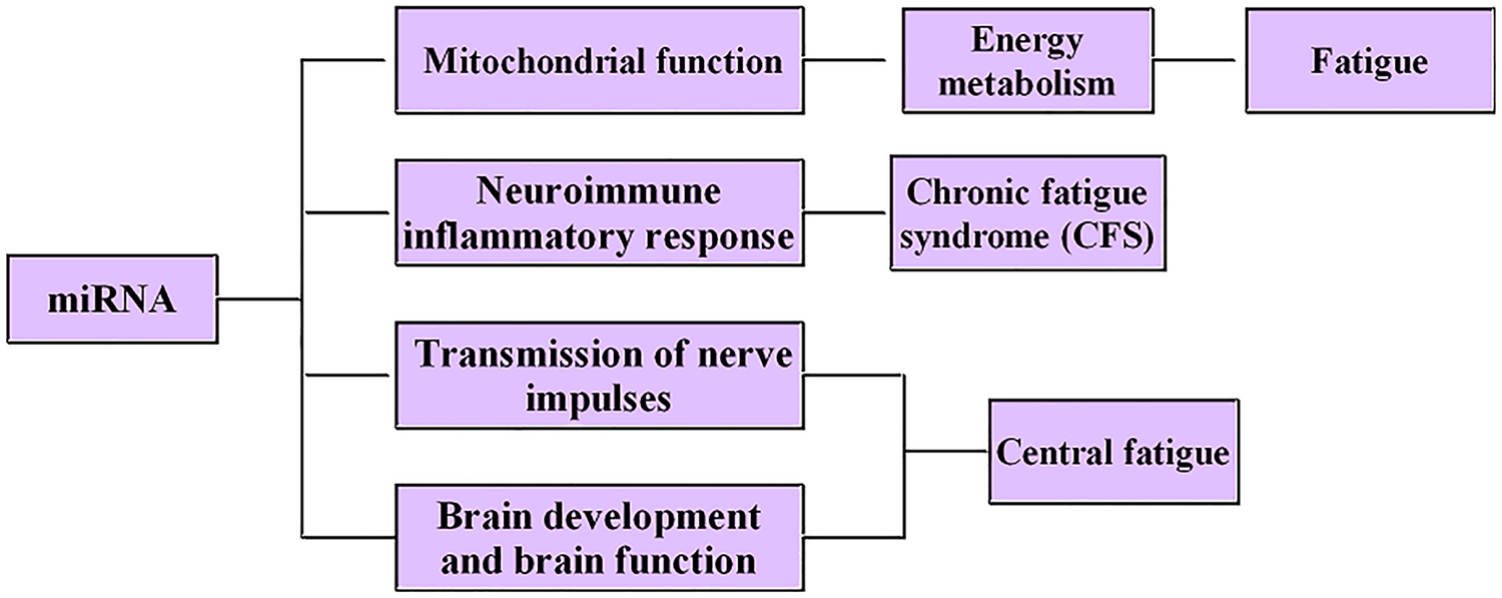
The role of miRNAs in the development of fatigue

MiRNAs are involved in the development of chronic fatigue syndrome (CFS) by regulating immune inflammatory response. For example, hsa-miR-99b and hsa-miR-330-3P in B cells; miR-21 in CD8^+^T cells; hsa-miR-99b, hsa-miR-330-3P, miR-21, miR-17-5p, miR-10a, miR-103, miR-152, miR-146a, miR-106, miR-223, mi R-191, hsa-miR-30c and hsa-miR-126 in NK cells can be effective biomarkers of CFS. Besides, miRNAs such as let-7b-5P, miR-148a-3P, miR-124-3P, miR-107-3P and miR-370-3P can regulate the transmission of nerve impulses and gene expression, so as to regulate the tolerance of the hypothalamus to acupuncture treatment and affect the fatigue of the body. MiR-124 can also promote the differentiation of neural progenitor cells into neurons, which plays an important role in regulating brain development and brain function (Fig. 2).

MiRNAs are involved in the occurrence and development of fatigue by regulating mitochondrial function and energy metabolism, regulating immune inflammatory reaction in the central nervous system (CNS), regulating nerve impulse transmission and gene expression and regulating brain development and brain function.

### MiRNAs, lncRNAs and circRNAs Can Form the Regulatory Network of ceRNAs

The regulation of gene expression is a complex interactive process, and transcriptome-level regulation is the key link in the gene regulatory network. Transcriptome generally refers to the collection of all transcripts in a cell, including messenger RNAs (mRNAs) and noncoding RNAs (ncRNAs); in a narrow sense, it refers to the collection of all mRNAs. NcRNAs are RNA molecules that do not translate into proteins, but participate in the regulation of various cellular and biological processes (Zhao et al. 2019). Besides miRNAs, ncRNAs also include lncRNAs, circRNAs and so on.

**Fig. 3:**
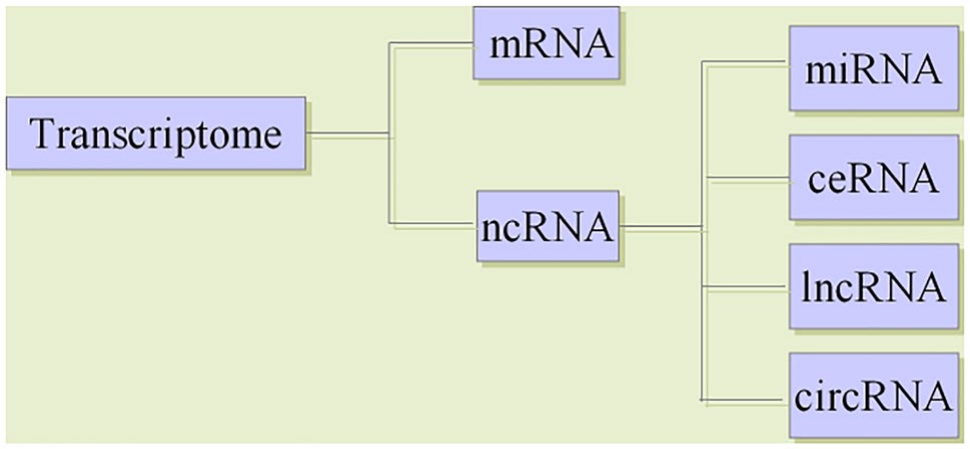
Classification of transcriptome RNAs

Long noncoding RNAs (lncRNAs) are a class of at least 200 nucleotides in length. Although the number of lncRNAs are much larger than that of miRNAs, the mechanism of regulating gene expression and cell function of lncRNAs is still unclear (Fang and Fullwood 2016; Jarroux et al. 2017). CircRNAs are endogenous ncRNAs with a covalently closed continuous loop structure (Chen et al. 2019; Chen and Zhao 2018). In recent years, studies have shown that circRNAs are widely expressed in tissues, saliva, blood and exosomes (Denzler et al. 2014) (Fig. 3).

Transcriptome RNAs includes mRNA and ncRNA. Besides miRNAs, ncRNAs also includes lncRNAs, circRNAs and so on. In 2011, Salmena et al. (2011) proposed the hypothesis of endogenous competitive RNA (ceRNA), which revealed a new mechanism of RNA interaction. This hypothesis posits that, in addition to gene silencing by binding to mRNAs, miRNAs can form a miRNA-mRNA network to regulate gene expression, and lncRNAs and circRNAs can regulate gene expression by competitively binding to the miRNA binding site MREs (Kartha and Subramanian 2014). CeRNAs are related to many biological processes, and the disruption of the balance between ceRNAs and miRNAs is crucial in the occurrence and development of diseases (Cesana et al. 2011).

### The Regulatory Role of miRNAs is the Core of the ceRNA Network

**Fig. 4:**
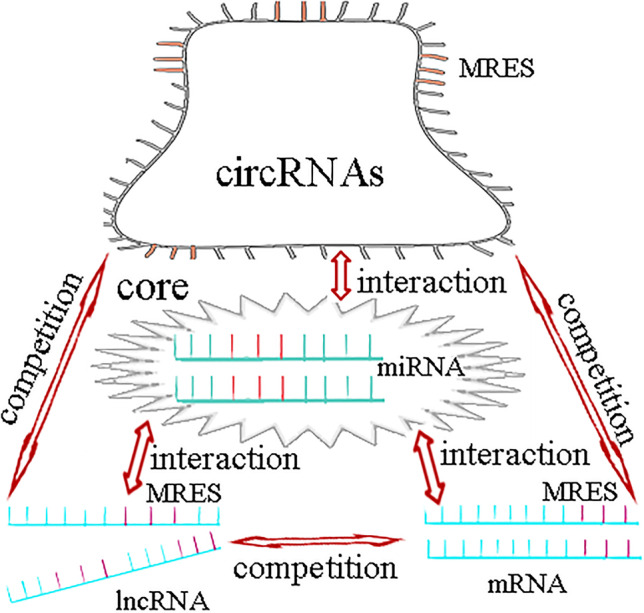
MiRNAs play a key role in the regulatory network of ceRNA

The research of ncRNA is developing rapidly, and it is a hot spot in current research (Baril et al. 2015). Research in the field of ncRNA has been advancing remarkably fast and remains a focus of intense research (Mallela and Nishikura 2012; Yang et al. 2013). After more than 20 years of extensive research, it is believed that at least 60% of the transcriptome is controlled by miRNAs. MiRNAs play a key role in the ncRNA regulatory network (Kole et al. 2012) (Fig. 4).

Previous studies have demonstrated that miRNAs can cause gene silencing by binding to mRNA. MiRNA-mRNA regulates gene expression through multiple networks. In addition to the traditional one-way miRNA regulation of mRNA function, lncRNAs and circRNAs can regulate gene expression by competitive binding to miRNA binding site MREs.

### The Correlation Between the ceRNA Regulatory Network and Fatigue

MiRNAs, lncRNAs and circRNAs play an important role in regulating brain function and brain diseases (Chen and Qin 2015; Andersen and Lim 2018; Hanan et al. 2017). It has been reported that ncRNAs are dynamically expressed in human brain, which has a precise spatiotemporal expression pattern and mediates a wide range of biological processes. The disorder of ncRNAs may not only lead to brain dysfunction, but also lead to mental disorders (Kang et al. 2011; Zhang et al. 2017; Hawrylycz et al. 2012; Guennewig and Cooper 2014).

Recent studies have shown that lncRNAs are highly expressed in the brain, and their roles in regulating brain development and function have been widely studied (Andersen and Lim 2018; Quan et al. 2017). Among them, many lncRNAs are functional lncRNAs that regulate brain development, including rhabdomyosarcoma 2-associated transcript (RMST) which is regulated by the repressor element-1-silencing transcription factor (REST) and is an indispensable factor in neurogenesis. RMST is brain-specific and highly expressed during the differentiation of dopaminergic neurons (Uhde et al. 2010). LncRNA NEAT1 gene knockout can effectively reduce the damage of dopaminergic neurons in vivo (Yan et al. 2018). Dopamine (DA) is an important excitatory neurotransmitter in the central nervous system, which can actively regulate emotions, improve memory and relieve fatigue (Leite et al. 2010; McMorris et al. 2018). Therefore, lncRNAs can participate in the occurrence and development of fatigue by regulating dopaminergic neurons. In addition, 10 ultra-long lncRNAs were detected in peripheral blood mononuclear cells of 44 CFS patients. The results showed that the levels of lncRNAs NTT, MIAT and EmX2OS in CFS patients were significantly higher than those in healthy controls. In addition, the levels of NTT and EmX2OS increased with the severity of the disease. This study revealed the function and potential diagnostic value of lncRNAs in CFS (Yang et al. 2018).

**Fig. 5:**
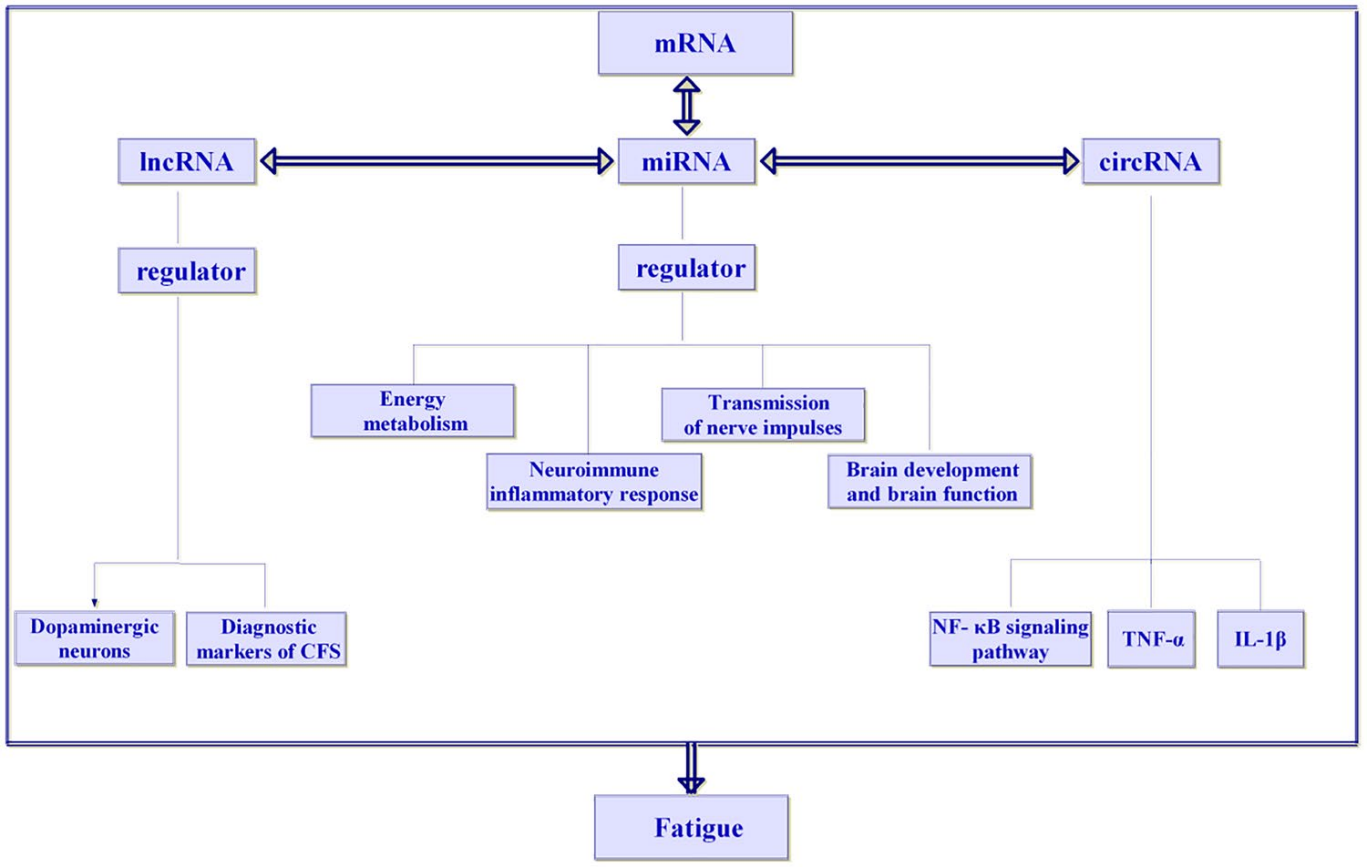
The regulatory network of ceRNA with miRNAs as the core is related to fatigue

In recent years, increasing evidence has shown that circRNAs are regulatory factors of neuroinflammation (Nuzziello and Liguori 2019). MALAT1 was recently reported to promote the inflammatory response in microglia via the MyD88/IRAK1/TRAF6 signalling pathway (Wang and Zhou 2018) and via miR-199b/IKKβ/NF-κB signalling, and to promote the production of proinflammatory cytokines (TNF-α and IL-1β) by acting as a ceRNA for miR-199b (Zhou et al. 2018). Neuroinflammatory reaction is closely related to fatigue (Capuron and Miller 2011). Proinflammatory cytokines, especially IL-1β, are the key to inducing fatigue (Lampa et al. 2012). High-intensity repeated exercise to the state of fatigue was shown to lead to an inflammatory reaction of the central nervous system, leading to significant upregulation of IL-1β and TNF-α in the brain of mice (Zhang 2015). Chronic fatigue syndrome (CFS) is characterized by activated immune inflammatory pathways, including increased pro-inflammatory cytokines and activation of NF-κB (Morris and Maes 2012). NF-κB can induce the transcription of TNF-α and IL-1β (Wu et al. 2019, 2020; Han et al. 2019). It can be concluded that the NF-κB pathway, TNF-α and IL-1β are closely related to fatigue. CircRNAs may participate in the occurrence and development of fatigue by regulating the NF-κB pathway, TNF-α and IL-1β (Fig. 5).

MiRNAs in ncRNA are closely related to the occurrence and development of fatigue, and lncRNAs and circRNAs are also related to the occurrence and development of fatigue. lncRNAs can participate in the occurrence and development of fatigue by regulating dopaminergic neurons. lncRNAs also have potential diagnostic value in the occurrence and development of CFS. The NF-κB signaling pathway, TNF-α and IL-1β are involved in the development of fatigue. The ceRNA hypothesis points out that in addition to the function of unidirectional miRNAs in regulating mRNAs, lncRNAs and circRNAs can regulate gene expression by competitive binding with miRNAs, forming a ceRNA regulatory network with miRNAs. Therefore, the regulatory network of ceRNAs with miRNAs as the core is closely related to fatigue.

## Conclusions

The regulation of gene expression is a complex interactive process, and transcriptome-level regulation is the key link in the gene regulatory network. The transcriptome includes mRNA and ncRNA. mRNA is a common research target in fatigue gene expression profiling. The miRNAs in ncRNAs are closely related to the occurrence and development of fatigue, and lncRNAs and circRNAs are related to the occurrence and development of fatigue. The ceRNA hypothesis posits that in addition to the function of unidirectional miRNAs in regulating mRNA, lncRNAs and circRNAs can regulate gene expression by competitive binding with miRNAs, forming a ceRNA regulatory network with miRNAs. Therefore, we suggest that the miRNA-centered ceRNA regulatory network is closely related to fatigue. At present, there are few studies on fatigue-related ncRNA microarrays, and most of the limited studies are on the miRNA microarray in ncRNAs. However, few genetic studies have been carried out on the pathogenesis of fatigue based on the ceRNA regulatory network. The pathogenesis of fatigue is complex, and the genetic research of fatigue is a hot spot. To elucidate the pathogenesis of fatigue, we need to find the key molecules of the regulatory network composed of related factors. Further exploration of the regulatory role of the miRNA-centered ceRNA regulatory network in the occurrence and development of fatigue will lead to a comprehensive, in-depth and new understanding of the essence of the fatigue gene regulatory network.

## Abbreviations

CFS: Chronic fatigue syndrome
CNS: Central nervous system
DNA: Deoxyribonucleic acid
lncRNAs: Long noncoding RNAs
mRNAs: Messenger RNAs
mtDNA: Mitochondrial DNA
ncRNAs: Noncoding RNAs
PBMC: Peripheral blood mononuclear cells
REST: Repressor element-1-silencing transcription
RMST: Rhabdomyosarcoma 2-associated transcript
RNAs: Ribonucleic acids

## References

[CR1] Andersen RE, Lim DA (2018). Forging our understanding of lncRNAs in the brain. Cell Tissue Res.

[CR2] Baril P, Ezzine S, Pichon C (2015). Monitoring the spatiotemporal activities of miRNAs in small animal models using molecular imaging modalities. Int J Mol Sci.

[CR3] Bhayani MK, Calin GA, Lai SY (2012). Functional relevance of miRNA sequences in human disease. Mutat Res.

[CR4] Brenu EW, Ashton KJ, Batovska J (2014). High-Throughput Sequencing of plasma Micro RNA in Chronic Fatigue Syndrome/Myalgic Encephalomyelitis. PLoS ONE.

[CR5] Brenu EW, Ashton KJ, van Driel M (2012). Cytotoxic lymphocyte microRNAs as prospective biomarkers for chronic fatigue syndrome/myalgic encephalomyelitis. J Affect Disord.

[CR6] Capuron L, Miller AH (2011). Immune system to brain signaling: neuropsychopharmacological implications. Pharmacol Ther.

[CR7] Cesana M, Cacchiarelli D, Legnini I (2011). A long noncoding RNA controls muscle differentiation by functioning as a competing endogenous RNA. Cell.

[CR8] Chen S, Zhao Y (2018). Circular RNAs: Characteristics, function, and role in human cancer. Histol Histopathol.

[CR9] Chen W, Qin C (2015). General hallmarks of microRNAs in brain evolution and development. RNA Biol.

[CR10] Chen X, Yang T, Wang W (2019). Circular RNAs in immune responses and immune diseases. Theranostics.

[CR11] Correia de Sousa M, Gjorgjieva M, Dolicka D (2019). Deciphering miRNAs Action through miRNA Editing. Int J Mol Sci.

[CR12] Costa V, Angelini C, De Feis I (2010). Uncovering the complexity of transcriptomes with RNA-Seq. J Biomed Biotechnol.

[CR13] Cui L, Ding Y, Feng Y (2016). MiRNAs are involved in chronic electroacupuncture tolerance in the rat hypothalamus. Mol Neurobiol.

[CR14] Denzler R, Agarwal V, Stefano J (2014). Assessing the ceRNA hypothesis with quantitative measurements of miRNA and target abundance. Mol Cell.

[CR15] El-Hattab AW, Craigen WJ (1863). Scaglia F (2017) Mitochondrial DNA maintenance defects. Biochim Biophys Acta Mol Basis Dis.

[CR16] Fang Y, Fullwood MJ (2016). Roles, Functions, and Mechanisms of Long Non-coding RNAs in Cancer. Genom Proteom Bioinf.

[CR17] Follert P, Cremer H, Béclin C (2014). MicroRNAs in brain development and function: A matter of flexibility and stability. Front Mol Neurosci.

[CR18] Frampton D, Kerr J, Harrison TJ (2011). Assessment of a 44 gene classifier for the evaluation of chronic fatigue syndrome from peripheral blood mononuclear cell gene expression. PLoS ONE.

[CR19] Guennewig B, Cooper AA (2014). The central role of noncoding RNA in the brain. Int Rev Neurobiol.

[CR20] Ha M, Kim VN (2014). Regulation of microRNA biogenesis. Nat Rev Mol Cell Biol.

[CR21] Halley SL, Marshall P, Siegler JC (2019). Effect of ischemic preconditioning and changing inspired O2 fractions on neuromuscular function during intense exercise. J Appl Physiol (1985).

[CR22] Han CJ, Zheng JY, Sun L (2019). The oncometabolite 2-hydroxyglutarate inhibits microglial activation via the AMPK/mTOR/NF-κB pathway. Acta Pharmacol Sin.

[CR23] Hanan M, Soreq H, Kadener S (2017). CircRNAs in the brain. RNA Biol.

[CR24] Haroon E, Raison CL, Miller AH (2012). Psychoneuroimmunology meets neuropsychopharmacology: translational implications of the impact of inflammation on behavior. Neuropsychopharmacology.

[CR25] Hawrylycz MJ, Lein ES, Guillozet-Bongaarts AL (2012). An anatomically comprehensive atlas of the adult human brain transcriptome. Nature.

[CR26] He M, Lu Y, Xu S (2014). MiRNA-210 modulates a nickel-induced cellular energy metabolism shift by repressing the iron-sulfur cluster assembly proteins ISCU1/2 in Neuro-2a cells. Cell Death Dis.

[CR27] Helliwell AM, Sweetman EC, Stockwell PA (2020). Changes in DNA methylation profiles of myalgic encephalomyelitis/chronic fatigue syndrome patients reflect systemic dysfunctions. Clin Epigenetics.

[CR28] Jarroux J, Morillon A, Pinskaya M (2017). History, discovery, and classification of lncRNAs. Adv Exp Med Biol.

[CR29] Kang HJ, Kawasawa YI, Cheng F (2011). Spatio-temporal transcriptome of the human brain. Nature.

[CR30] Kartha RV, Subramanian S (2014) Competing endogenous RNAs (ceRNAs): new entrants to the intricacies of gene regulation. Front Genet 5:8. 10.3389/fgene.2014.00008 10.3389/fgene.2014.00008 PMC3906566 24523727

[CR31] Kole R, Krainer AR, Altman S (2012). RNA therapeutics: Beyond RNA interference and antisense oligonucleotides. Nat Rev Drug Discov.

[CR32] Konovalova J, Gerasymchuk D, Parkkinen I (2019). Interplay between MicroRNAs and Oxidative Stress in Neurodegenerative Diseases. Int J Mol Sci.

[CR33] Lampa J, Westman M, Kadetoff D (2012). Peripheral inflammatory disease associated with centrally activated IL-1 system in humans and mice. Proc Natl Acad Sci USA.

[CR34] Leavitt VM, DeLuca J (2010). Central fatigue: issues related to cognition, mood and behavior, and psychiatric diagnoses. PMR.

[CR35] Leite LH, Rodrigues AG, Soares DD (2010). Central fatigue induced by losartan involves brain serotonin and dopamine content. Med Sci Sports Exerc.

[CR36] Leung CS, Lu S, Li J (2018). Deciphering the Role of microRNAs in Regulation of Immune Surveillance, Self-Tolerance and Allograft Transplant Outcome. Curr Stem Cell Res Ther.

[CR37] Li F, Han CX, Wu FZ et al (2016) Modern research on fatigue. Chinese science: life science 46(8):903–912.https://kns.cnki.net/kcms/detail/detail.aspx?dbcode=CJFD&dbname=CJFDLAST2016&filename=JCXK201608002&v=rWECW9y1LdHfDYHoWobttvLcs2WtFTU7aoVqVb7jbvL5lMyUsEi73b0%25mmd2BEqV8taBS

[CR38] Lievesley K, Rimes KA, Chalder T (2014). A review of the predisposing, precipitating and perpetuating factors in Chronic Fatigue Syndrome in children and adolescents. Clin Psychol Rev.

[CR39] Mallela A, Nishikura K (2012). A-to-I editing of protein coding and noncoding RNAs. Crit Rev Biochem Mol Biol.

[CR40] Matsuyama H, Suzuki HI (2019). Systems and Synthetic microRNA Biology: From Biogenesis to Disease Pathogenesis. Int J Mol Sci.

[CR41] McMorris T, Barwood M, Corbett J (2018). Central fatigue theory and endurance exercise: Toward an interoceptive model. Neurosci Biobehav Rev.

[CR42] Morris G, Maes M (2012). Increased nuclear factor-κB and loss of p53 are key mechanisms in Myalgic Encephalomyelitis/chronic fatigue syndrome (ME/CFS). Med Hypotheses.

[CR43] Nguyen CB, Alsøe L, Lindvall JM (2017). Whole blood gene expression in adolescent chronic fatigue syndrome: an exploratory cross-sectional study suggesting altered B cell differentiation and survival. J Transl Med.

[CR44] Norheim KB, Jonsson G, Omdal R (2011) Biological mechanisms of chronic fatigue. Rheumatology (Oxford) 50(6):1009–1018. 10.1093/rheumatology/keq454 10.1093/rheumatology/keq454 21285230

[CR45] Nuzziello N, Liguori M (2019). The MicroRNA Centrism in the Orchestration of Neuroinflammation in Neurodegenerative Diseases. Cells.

[CR46] Petri R, Malmevik J, Fasching L et al (2014) MiRNAs in brain development. Exp Cell Res 321(1):84–89. 10.1016/j.yexcr.2013.09.022 10.1016/j.yexcr.2013.09.022 24099990

[CR47] Petty RD, Mccarthy NE, Dieu R (2016). MicroRNAs hsa-miR-99b, hsa-miR-330, hsa-miR-126 and hsa-mi R-30c: potential Diagnostic Biomarkers in Natural Killer (NK) Cells of patients with Chronic Fatigue Syndrome (CFS)/Myalgic Encephalomyelitis (ME). PLoS ONE.

[CR48] Pu M, Chen J, Tao Z (2019). Regulatory network of miRNA on its target: coordination between transcriptional and post-transcriptional regulation of gene expression. Cell Mol Life Sci.

[CR49] Quan Z, Zheng D, Qing H (2017). Regulatory Roles of Long Non-Coding RNAs in the Central Nervous System and Associated Neurodegenerative Diseases. Front Cell Neurosci.

[CR50] Rajeevan MS, Dimulescu I, Murray J (2015). Pathway-focused genetic evaluation of immune and inflammation related genes with chronic fatigue syndrome. Hum Immunol.

[CR51] Rupp T, Mallouf Tle R, Perrey S (2015). CO2 Clamping, Peripheral and Central Fatigue during Hypoxic Knee Extensions in Men. Med Sci Sports Exerc.

[CR52] Salmena L, Poliseno L, Tay Y (2011). A ceRNA hypothesis: the Rosetta Stone of a hidden RNA language?. Cell.

[CR53] Slota JA, Booth SA (2019). MicroRNAs in Neuroinflammation: Implications in Disease Pathogenesis, Biomarker Discovery and Therapeutic Applications. Noncoding RNA.

[CR54] Soltanzadeh-Yamchi M, Shahbazi M, Aslani S (2018). MicroRNA signature of regulatory T cells in health and autoimmunity. Biomed Pharmacother.

[CR55] Stavast CJ, Erkeland SJ (2019). The Non-Canonical Aspects of MicroRNAs: Many Roads to Gene Regulation. Cells.

[CR56] Uhde CW, Vives J, Jaeger I (2010). Rmst is a novel marker for the mouse ventral mesencephalic floor plate and the anterior dorsal midline cells. PLoS ONE.

[CR57] Wang LQ, Zhou HJ (2018). LncRNA MALAT1 promotes high glucose-induced inflammatory response of microglial cells via provoking MyD88/IRAK1/TRAF6 signaling. Sci Rep.

[CR58] Watanabe Y (2008) Preface and mini-review: fatigue science for human health. Fatigue Science for Human Health. Edited by: Watanabe Y, Evengård B, Natelson BH, Jason LA, Kuratsune H, New York: Springer, 5–11

[CR59] Wu H, Liu H, Zhao X (2020). IKIP Negatively Regulates NF-κB Activation and Inflammation through Inhibition of IKKα/β Phosphorylation. J Immunol.

[CR60] Wu J, Niu P, Zhao Y (2019). Impact of miR-223-3p and miR-2909 on inflammatory factors IL-6, IL-1ß, and TNF-α, and the TLR4/TLR2/NF-κB/STAT3 signaling pathway induced by lipopolysaccharide in human adipose stem cells. PLoS ONE.

[CR61] Yamano E, Sugimoto M, Hirayama A (2016). Index markers of chronic fatigue syndrome with dysfunction of TCA and urea cycles. Sci Rep.

[CR62] Yan W, Chen ZY, Chen JQ et al (2018) LncRNA NEAT1 promotes autophagy in MPTP-induced Parkinson’s disease through stabilizing PINK1 protein. Biochem Biophys Res Commun 496(4):1019–1024. 10.1016/j.bbrc.2017.12.149 10.1016/j.bbrc.2017.12.149 29287722

[CR63] Yang CA, Bauer S, Ho YC (2018). The expression signature of very long non-coding RNA in myalgic encephalomyelitis/chronic fatigue syndrome. J Transl Med.

[CR64] Yang Y, Zhou X, Jin Y (2013). ADAR-mediated RNA editing in non-coding RNA sequences. Sci China Life Sci.

[CR65] Zhang XQ, Wang ZL, Poon MW (2017). Spatial-temporal transcriptional dynamics of long non-coding RNAs in human brain. Hum Mol Genet.

[CR66] Zhang XX (2015) Neuroinflammatory mechanism of exercise fatigue impairing learning and memory ability in rats. Taiyuan University of Technology. https://kns.cnki.net/kcms/detail/detail.aspx?dbcode=CMFD&dbname=CMFD201502&filename=1015603661.nh&v=fP3ZOaOGHJvIyePi%25mmd2BeII%25mmd2BlmnO%25mmd2FItn2Ob7bD7NVoXEXowUAgDs0fmx2sO7dEH0bX5

[CR67] Zhao B, Chen Y, Hu S (2019). Systematic analysis of non-coding RNAs involved in the angora rabbit (oryctolagus cuniculus) hair follicle cycle by RNA sequencing. Front Genet.

[CR68] Zhou HJ, Wang LQ, Wang DB (2018). Long noncoding RNA MALAT1 contributes to inflammatory response of microglia following spinal cord injury via the modulation of a miR-199b/IKK_/NF-_B signaling pathway. Am J Physiol Cell Physiol.

